# Potential contribution of the gut microbiota to the development of portal vein thrombosis in liver cirrhosis

**DOI:** 10.3389/fmicb.2023.1217338

**Published:** 2023-10-27

**Authors:** Xin-yu Huang, Ying-hui Zhang, Shi-yu Yi, Lei Lei, Tao Ma, Rui Huang, Lan Yang, Zhen-mao Li, Di Zhang

**Affiliations:** Department of Gastroenterology and Hepatology, Sichuan Provincial People's Hospital, School of Medicine, University of Electronic Science and Technology of China, Chengdu, China

**Keywords:** liver cirrhosis, portal vein thrombosis, gut microbiota, coagulation disorders, *Bacteroides*

## Abstract

**Background:**

Portal vein thrombosis (PVT) is a serious complication of liver cirrhosis (LC) and is closely related to gut homeostasis. The study aimed to investigate the composition of gut microbiota and its putative role in PVT development in LC.

**Methods:**

33 patients with LC admitted between January 2022 and December 2022 were enrolled in this study. Based on imaging findings, they were categorized into LC without PVT (*n* = 21) and LC with PVT (*n* = 12) groups. Fecal samples were collected from each participant and underwent 16S rDNA sequencing.

**Results:**

D-Dimer and platelet elevations were the main clinical features of LC with PVT. The alpha and beta diversity of the gut microbiota in LC with PVT group was found to be significantly higher compared to the control group. The structure of the gut microbiota was significantly different between the two groups. Based on LEfSe data, the genera *Akkermansia*, *Eubacterium hallii group, Fusicatenibacter*, and *Anaerostipes* were enriched in the LC with PVT, while *Enterococcus, Weissella, Bacteroides,* and *Subdoligranulum* were enriched in those of the LC subjects. Changes in microbiota structure result in significant differences in gut microbiota metabolism between the two groups. Altered levels of the microbiota genera were shown to be correlated with coagulation factor parameters. In animal experiments, the addition of *Bacteroides* reversed the CCl_4_-induced PVT.

**Conclusion:**

Liver cirrhosis with PVT led to a disorder in the gut microbiota, which was characterized by an increase in pathogenic bacteria and a decrease in beneficial bacteria. Furthermore, modulating the gut microbiota, especially *Bacteroides*, may be a promising therapeutic approach to reduce the progression of PVT in LC.

## Introduction

Portal vein thrombosis (PVT) is an increasingly recognized complication of liver cirrhosis (LC). The prevalence of PVT has been found to increase in parallel with the severity of cirrhosis. It is observed to be 10% in patients with compensated cirrhosis (Tsochatzis et al., 2010), 7.4–17% in those with advanced cirrhosis (Child-Pugh B/C; Violi et al., 2019), and even up to 26% in liver transplant (LT) candidates (Francoz et al., 2012; Senzolo et al., 2021). PVT reportedly exerts a further increase in resistance to portal blood flow, leading to worsening portal hypertension, which results in an increased risk of gastrointestinal bleeding (Amitrano et al., 2002) and decompensation associated with portal hypertension (Senzolo et al., 2019), especially in complete PVT.

The development of PVT in cirrhosis is believed to have a multifactorial pathogenesis and is mainly due to changes in the different components of Virchow’s triad, including hypercoagulability, decreased portal vein flow, and damage to the vessel wall (Intagliata et al., 2019). The gut microbiota represents the most extensive population of microorganisms within the human body, and it directly interacts with the liver through the hepatointestinal axis (Baffy, 2019). The central role of the microbiota in a variety of chronic liver diseases has been established. Dysregulation of the gut microbiota may influence the intensity of liver inflammation and fibrosis by engaging in multiple interactions with the host immune system (Baffy, 2019). Bacterial translocation and bacterial products (such as endotoxins) lead to hepatic encephalopathy and spontaneous bacterial peritonitis (Bellot et al., 2013). Microflora imbalance may be involved in portal hypertension (Violi et al., 2023); however, a correlation between PVT occurrence and gut microflora has not been reported. Theoretically, PVT is closely related to dysbiosis of gut microbiota. We hypothesized that the presence of PVT in patients with cirrhosis can lead to disturbances in the gut microbiota, which aggravate the progression of cirrhosis and PVT.

Thus, we utilized 16S rRNA gene sequencing to compare and characterize the microbial community composition in patients with cirrhosis with and without PVT. Additionally, we conducted an analysis of the potential correlation between gut microbiota and coagulation function. The purpose of this study was to investigate the relationship between PVT development and gut microbiota and to provide a theoretical basis for improving gut dysbiosis in the treatment of PVT.

## Methods

### Study design and patients

Consecutive patients with LC were recruited for this prospective observational study, which was conducted between January 2022 and December 2022. The diagnosis of LC was established by evaluating clinical, laboratory, and radiological data, and/or conducting liver biopsies. The diagnosis of PVT followed the consensus guidelines for managing PVT in LC patients (2020, Shanghai), in brief the diagnosis of PVT relies mainly on imaging (liver vascular ultrasound, CT or MRI; Hepatobiliary Disease Study Group et al., 2021). The inclusion criteria required patients to be between 18 and 70 years of age, with well-characterized cirrhosis that was confirmed by compatible clinical, imaging, liver transient elastography, and laboratory values. Patients were excluded from the study if they were on anticoagulation therapy at the time of enrollment, had hepatocellular carcinoma (HCC), pregnant, prior trans jugular intrahepatic portosystemic shunt (TIPS), prior liver transplant, or systemic neoplasia.

### Ethic statement

This research was conducted in compliance with the tenets of the Declaration of Helsinki. The Ethics Committee of Sichuan Provincial People’s Hospital approved the study (No. SCCT2021-525), and all patients provided informed consent prior to participation.

### Fecal samples

A standardized method was used to collect fecal samples. Samples were transported to the laboratory within 2 h of collection along with an ice pack. The samples were promptly frozen and reserved at −80°C until the time of analysis.

### DNA extraction and microbiome profiling

The QIAamp Fast DNA Stool Mini Kit (Qiagen, Germany) was used to extract microbial DNA from fecal samples, following the manufacturer’s instructions. For PCR amplification of the bacterial 16S ribosomal RNA genes, primers 341F (5′-CCTACGGGRSGCAGCAG-3′) and 806R (5′-GGACTACVVGGGTATCTAATC-3′) were used to target the V3-V4 regions of the genes. Amplicons were extracted from 2% agarose gels and purified using the AxyPrep DNA Gel Extraction Kit (Axygen Biosciences, Union City, CA, United States) according to the manufacturer’s instructions and quantified using Qubit®2.0 (Invitrogen, United States). All quantified amplicons were pooled to equalize concentrations for sequencing using Illumina NovaSeq (Illumina, Inc., CA, United States). Using cutadapt in Qiime2 (v2022.2) to remove primer sequences from the data. DNA extraction, Library construction, and sequencing were conducted at Realbio Genomics Institute (Shanghai, China).

### Bioinformatics analysis

The DADA2 plug-in in Qiime2 (v2022.2) software was used to filter, denoise, merged and nonchimeric the sequence after the removal of primes, and the Amplicon Sequence Variant or Feature sequence (ASV) was formed. The Feature representative sequences were compared with the Silva database (v138) to obtain the species classification of each feature, and then subsequent analysis was conducted according to the species abundance table.

Alpha-diversity metrics, such as Chao1 richness estimator, observed species, Faith’s PD, and Good’s coverage were calculated using the ASV table in QIIME2, and visualized as box plots. Beta diversity analysis was performed to investigate the structural variation of microbial communities across samples using Jaccard metrics, Bray–Curtis metrics, and UniFrac distance metrics, and visualized via principal coordinate analysis, non-metric multidimensional scaling, and unweighted pair-group method with arithmetic means hierarchical clustering. We employed linear discriminant analysis (LDA) of effect size (LEfSe) to identify significantly different taxa between the two groups (the default screening condition was LDA ≥ 2). Microbial functions were predicted by PICRUSt2 upon MetaCyc and KEGG databases.

### Animal studies

Male Wistar rats were purchased from Yangzhou University Animal Medicine Center (SCXK 2022-0009). Rats were randomly divided into three groups (five rats per group): Control, CCl_4_, and CCl_4_+ *Bacteroides* group. Animals in all groups were provided a standard commercial rodent diet and were maintained on a 12-h light/dark cycle at constant temperature and humidity. All experimental procedures were approved by the Ethical Committee of Sichuan Provincial People’s Hospital. In addition, the National Institutes of Health guide for the care and use of laboratory animals was strictly followed.

Rats in the CCl_4_ group or CCl_4_+ *Bacteroides* group were gavaged with CCl_4_ dissolved (MACKLIN, C805329) in olive oil (40% v/v, 3 mL/kg) twice per week for 12 weeks. During this 12-week treatment course, rats in the CCl_4_+ *Bacteroides* group were gavaged with 1*10^6^ pieces/mL of bacterial (BeNa Culture, BNCC336948) solution (5 mL/day). At weeks 8, 9, 10, 11, and 12, portal blood flow and thrombosis were detected in rats using small animal ultrasound. The animals were euthanized at 12 weeks under deep anesthesia by intraperitoneal injection with an overdose of pentobarbital sodium (200 mg/kg) to obtain their samples for biochemical and molecular analyses.

### Statistical analysis

For bioinformatics statistical analysis, an independent sample *t*-test was used to analyze normally distributed variables. Non-normally distributed variables were analyzed using the Mann–Whitney U test. A value of *p* < 0.05 was considered statistically significant. The α-diversity index analysis was performed using Qiime 2 software. Each index of Alpha diversity was analyzed by rank sum test. If two sets of samples are compared, wilcox.test function in R is used, and if more than two sets of samples are compared, the kruskal.test function in R is used.

For clinical data analysis, the statistical analysis was conducted with SPSS software. Continuous variables that exhibited normal distribution were presented as mean ± SD, and the *t*-test was utilized to compare the differences. We utilized nonparametric tests to compare continuous variables that were not normally distributed. Categorical variables were represented using percentages and integers, and groups were compared using Fisher’s exact test. Statistical significance was determined at a *p* value of <0.05.

## Results

### Clinical characteristics

Twelve patients with liver cirrhosis with PVT and 21 patients without PVT as controls were included in this cross-sectional study. The demographic characteristics and clinical parameters of the participants are presented in Table 1. The LC with PVT group had higher levels of platelet and D-Dimer compared to the control group. However, there were no statistically significant differences in body mass index, sex, other laboratory indicators, primary etiology of cirrhosis, or Child-Pugh grade between the two groups (Table 1).

**Table 1:** Demographic and laboratory characteristics of the study sample.

Features	LC (*n* = 21)	LC + PVT (*n* = 12)	*p* value
Age (years)	57.14 ± 8.99	52.83 ± 10.84	0.2283
Gender (male, %)	8, 38.1%	3, 25%	0.7062
BMI (kg/m^2^)	22.97 ± 3.14	24.09 ± 5.29	0.4475
Primary etiology			0.3618
Viral	16, 76.2%	8, 66.7%	
Alcohol	1, 9.5%	2, 16.7%	
Immunity	3, 14.3%	1, 8.3%	
Other	0, 0.0%	1, 8.3%	
AST (μ/L)	59.95 ± 78.51	44.67 ± 14.44	0.5118
ALT (μ/L)	33.46 ± 7.19	31.33 ± 15.51	0.5931
Albumin (g/L)	33.41 ± 9.26	31.94 ± 4.85	0.6137
TB (μmol/L)	29.53 ± 37.86	37.79 ± 28.77	0.5178
SCr (μmol/L)	64.40 ± 17.02	61.99 ± 9.35	0.6552
WBC (×10^9^/L)	3.21 ± 1.45	4.36 ± 2.16	0.0764
Platelets (×10^9^/L)	48.67 ± 15.60	92.83 ± 98.02	**0.0495** ^*^
PT (s)	14.79 ± 2.92	15.13 ± 2.18	0.7258
PT% (%)	60.35 ± 18.38	54.53 ± 12.74	0.3399
PT-INR	1.35 ± 0.29	1.40 ± 0.21	0.6098
FIB (g/L)	1.72 ± 0.66	1.68 ± 0.63	0.8418
D-Dimer (mg/L)	2.03 ± 1.76	3.77 ± 1.87	**0.0122** ^*^
Ascites (mild/moderate/severe)	11/7/3	5/6/1	0.6242
Blood ammonia (μmol/L)	69.45 ± 21.41	68.78 ± 35.37	0.9462
Child-Pugh grade (A/B/C)	9/8/4	3/6/3	0.5909

BMI, Body mass index; ALT, Alanine transaminase; AST, Aspartate transaminase; PT, Prothrombin time; TB, Total bile acid; SCr, Serum creatinine; INR, International normalized ratio; FIB, Fibrinogen; and WBC, White blood cell. ^*^p < 0.05.

The bold values represent statistical significance.

### Dynamics of gut microbiota in LC patients with PVT

**Figure 1:**
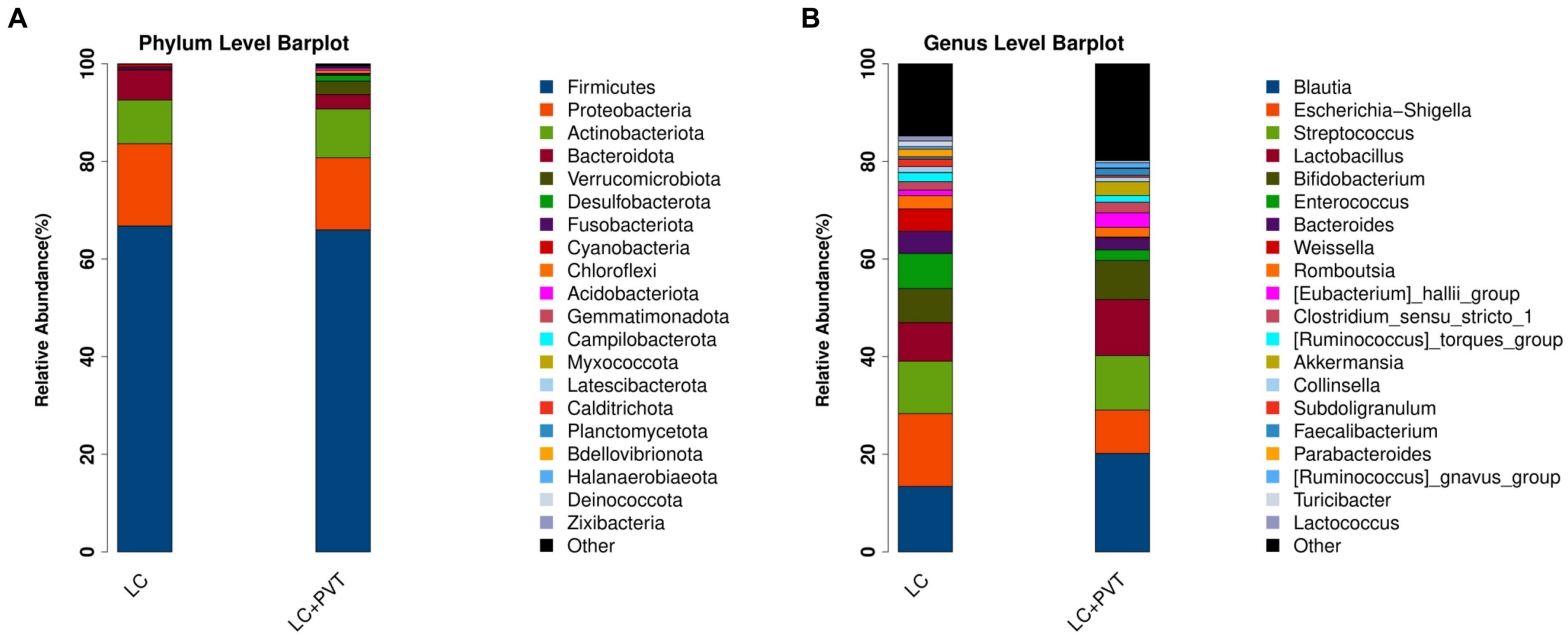
Different profiles of the gut microbiota between the LC with PVT and LC controls. **(A)** Top 20 abundant phyla. **(B)** Top 20 abundant genera.

**Figure 2:**
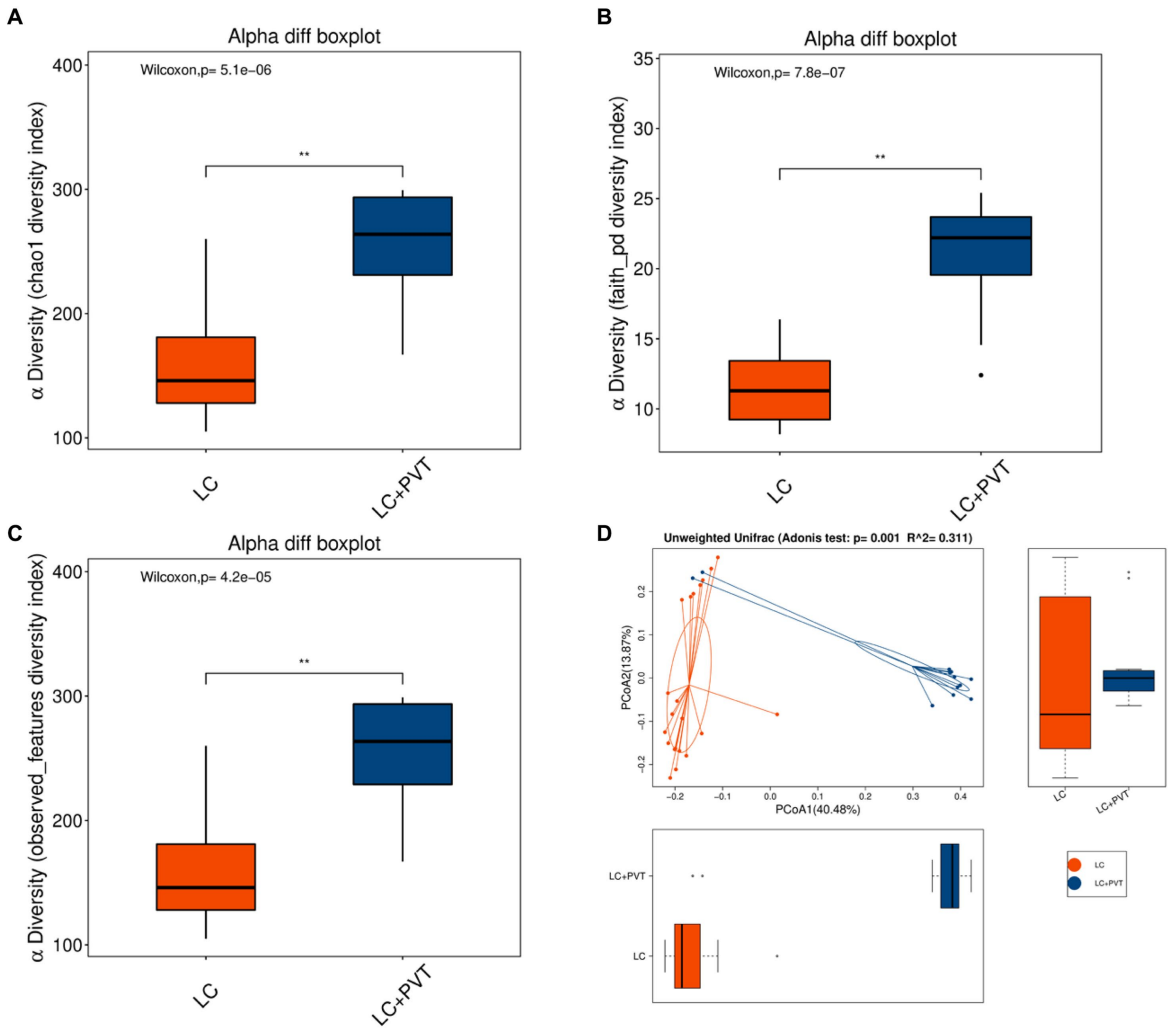
Gut microbiota alpha and beta diversity indices in LC with PVT and LC controls. The box plots depict differences in microbiome diversity indices between two groups using the **(A)** Chao1, **(B)** Phylogenetic Diversity (PD) whole-tree distance index, and **(C)** Observed species. **(D)** The level of similarity between the detected gut microbiota communities was assessed via an unweighted analysis of similarity (ANOSIMs).

Initially, we examined the species composition and abundance of gut microorganisms in both groups at both the phylum and genus levels. *Firmicutes*, *Proteobacteria*, *Actinobacteria*, and *Bacteroidetes* collectively accounted for >95% of the relative abundance in both groups. Compared to the control group, the abundances of *Firmicutes* (66.8 vs. 66.0%) and *Proteobacteria* (16.9 vs. 14.8%) in the LC with PVT group were relatively low, whereas the abundances of *Actinobacteria* (9.0 vs. 10.0%) and *Bacteroidetes* (6.1 vs. 2.9%) were higher (Figure 1A). At the genus level, the five most abundant bacterial taxa in the LC group were *Escherichia-Shigella* (14.9%), *Blautia* (13.4%), *Streptococcus* (10.7%), *Lactobacillus* (7.9%), and *Enterococcus* (7.2%). However, in the LC with PVT group, *Blautia* (20.2%), *Lactobacillus* (11.4%), *Streptococcus* (11.2%), *Escherichia-Shigella* (8.9%), and Bifidobacterium (8.0%) was detected (Figure 1B). When comparing the alpha diversity of the gut microbiota between the two groups, we found that the Chao1, observed species, and PD diversity indices were significantly higher in the group with LC and PVT than in the control group. The PCoA of beta diversity based on unweighted UniFrac revealed significant differences in community composition between the two groups (Figures 2A–D).

**Figure 3:**
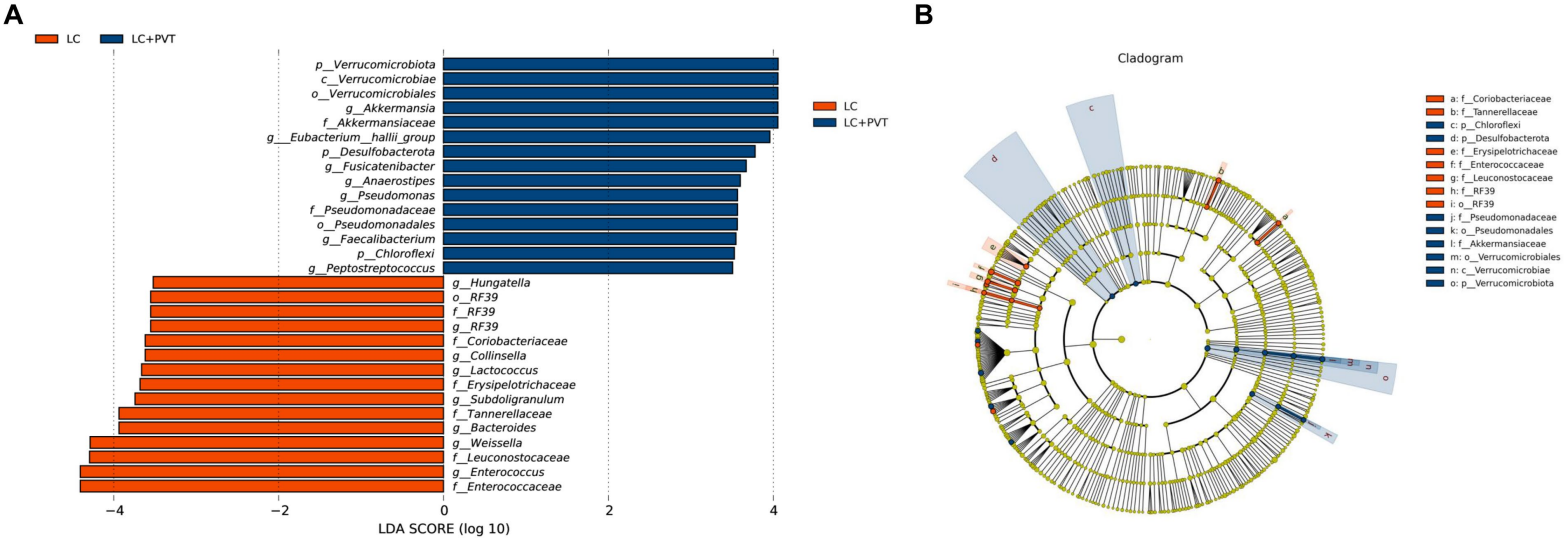
Taxonomic differences in the gut microbiota exhibited by LC patients with and without PVT. **(A)** The histogram of the LDA scores presented the relative abundance of the main bacterial in the two groups. **(B)** LEfSe results in a cladogram of gut microbiota between the two groups.

To identify differences in gut microbiota between the two groups, LDA and LEfSe analyses were conducted. Using a logarithmic LDA score with a cutoff value of 2.0, we identified important taxonomic differences and presented the top 30 differential gut microbiota. Our results suggest that there was a remarkable difference in gut microbiota between the two groups. The highest relative abundances in LC with PVT were for *Akkermansia*, *Eubacterium hallii group, Fusicatenibacter*, *Anaerostipes*, *and Pseudomonas*. In contrast, *Enterococcus*, *Weissella*, *Bacteroides*, *Subdoligranulum*, *Lactococcus*, *Collinsella*, *RF39*, *and Hungatella* were enriched in LC controls (Figures 3A,B).

### Gut microbiota metabolic pathway analysis

**Figure 4:**
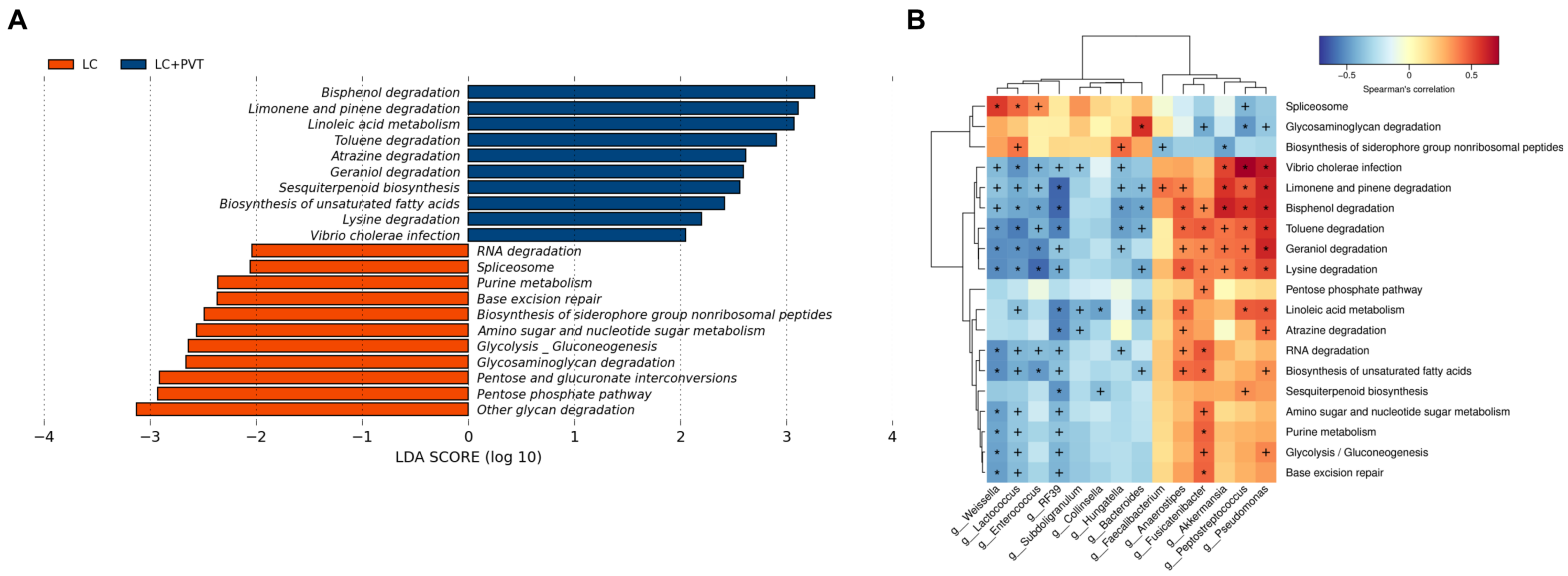
Gut microbiota metabolic pathway analysis. **(A)** KEGG metabolic pathway analysis. **(B)** Microbe-derived metabolite enrichment analysis results.

Kyoto Encyclopedia of Genes and Genomes (KEGG) pathways were analyzed using linear discriminant analysis (LDA) effect size (LEfSe) to predict the potential association between PVT and gut microbial metabolism. There were significant differences in gut microbial metabolism between the two groups; the top five candidate metabolic pathways in the LC group were other glycan degradation, pentose phosphate pathway, pentose and glucuronate interconversions, glycosaminoglycan degradation, and glycolysis/gluconeogenesis. The top five metabolic pathways in the LC with PVT group were bisphenol degradation, limonene and pinene degradation, linoleic acid metabolism, toluene degradation, and atrazine degradation (Figure 4A). We further analyzed the associations between the microbes and the derived metabolites (Figure 4B).

### Associations among gut microbiota and coagulation function

**Figure 5:**
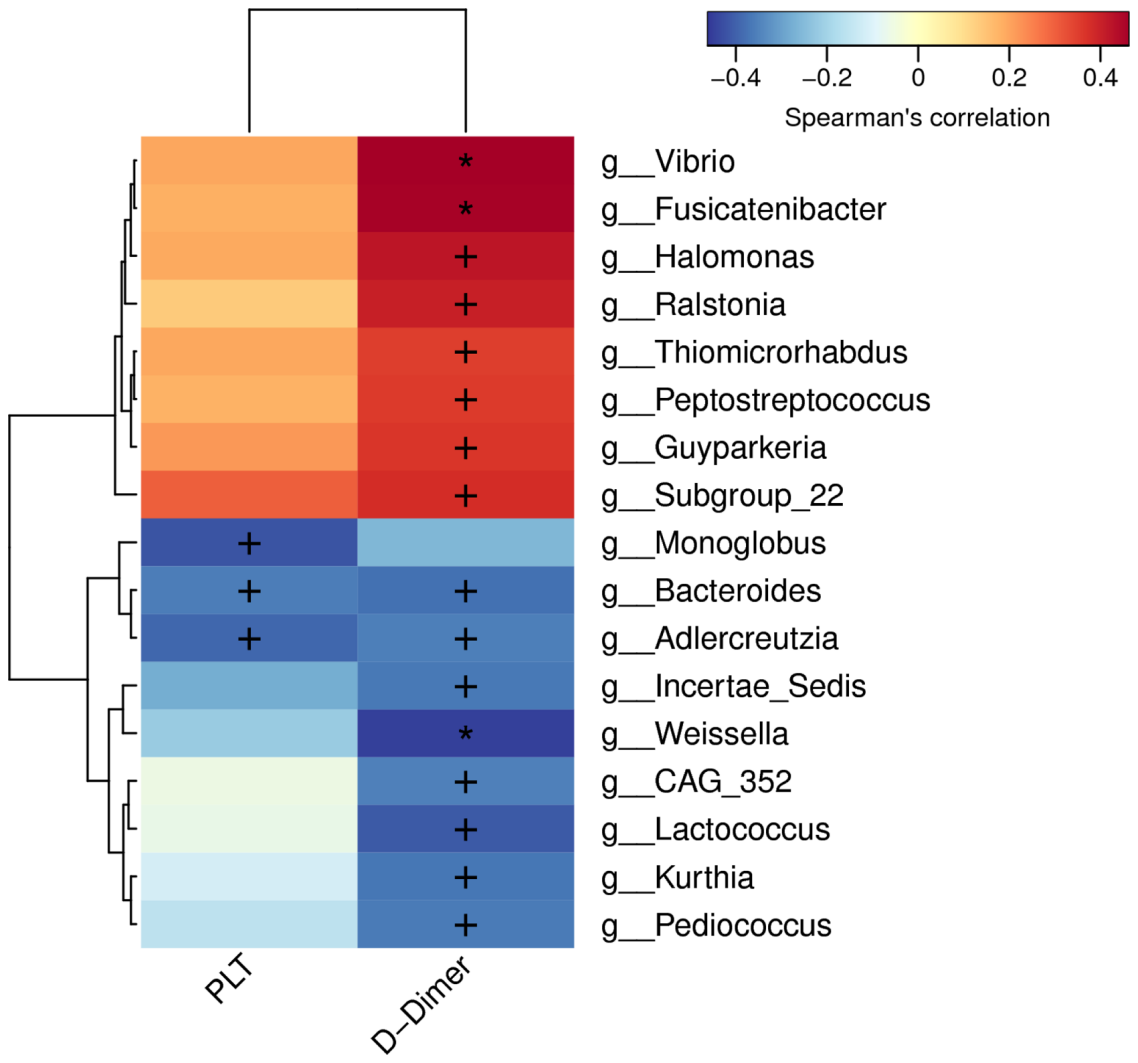
Correlations between differentially abundant microbiota genera and coagulation factor parameters.

We analyzed the potential correlations between differentially abundant gut microbiota and coagulation function parameters (PLT, PT, INR, FIB, and D-Dimer). PLT and D-Dimer were correlated with a partial abundant of gut microbiota. *Vibrio, Fusicatenibacter, Halomonas, Ralstonia, Thiomicrorhabdus, Peptostreptococcus, Guyparkeria, and Subgroup-22* significantly and actively correlated with D-Dimer levels, whereas *Bacteroides*, *Adlercreutzia, Incertae-Sedis, Weissella, CAG-352, Lactococcus, Kurthia*, *and Pediococcus* negatively correlated. *Monoglobus, Bacteroides, and Adlercreutzia* were significantly and negatively correlated with PLT (Figure 5).

### Bacteroides improve CCl4-induced PVT in Wistar rats

**Figure 6:**
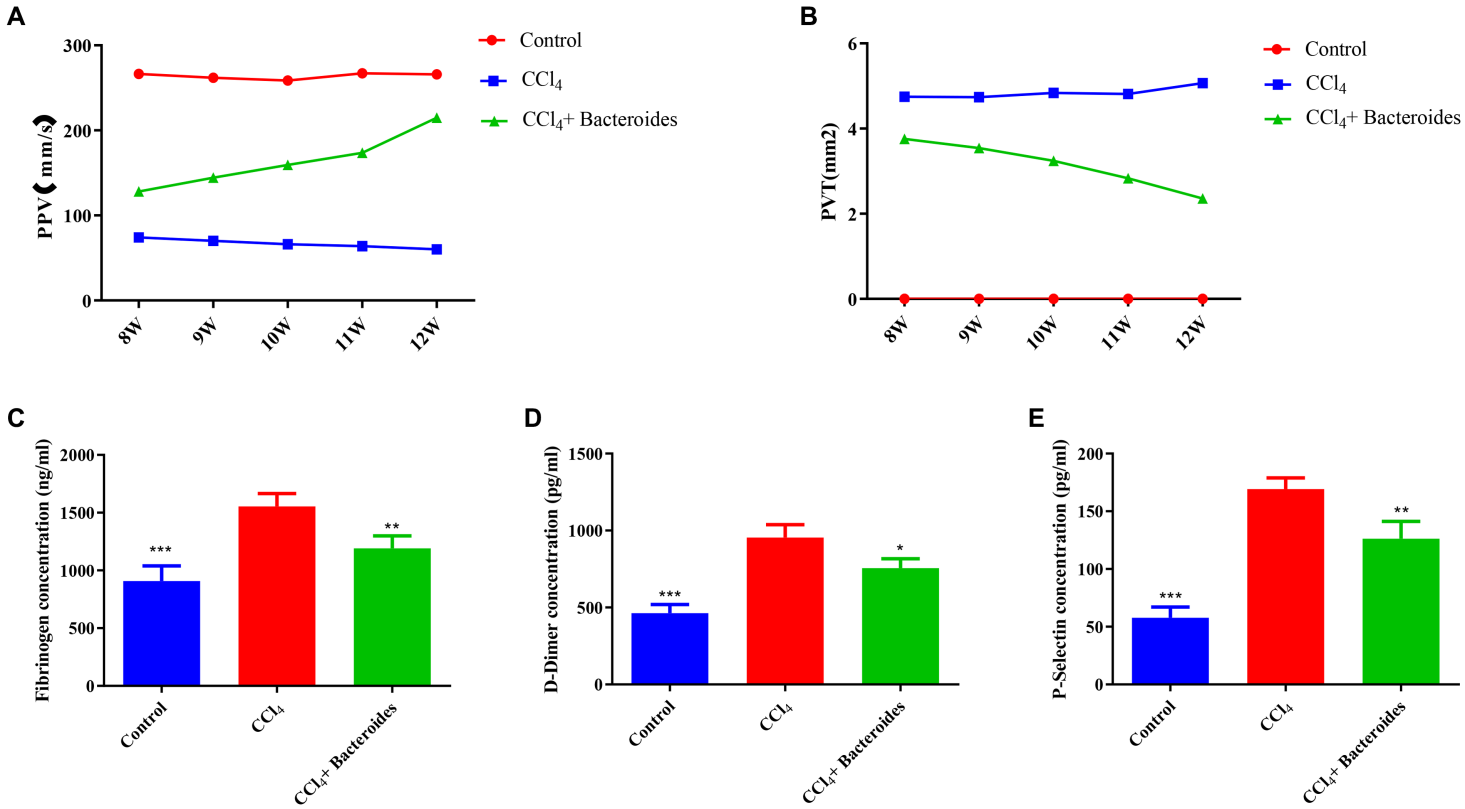
Bacteroides improve CCl4-induced PVT in Wistar rats. Using a small animal ultrasound instrument to detect **(A)** the blood flow velocity of portal vein, **(B)** the size of portal vein thrombosis. ELISA analysis of the fibrinogen **(C)**, D-Dimer **(D)**, and P-Selectin **(E)** in plasma.

The *Bacteroides*, a type of intestinal probiotic, is widely considered as source of novel beneficial candidates for attenuating inflammation (Tan et al., 2019; Guo et al., 2021). In our results, *Bacteroides* was significantly decreased in the PVT group, so we explored its effect on PVT by supplementing with *Bacteroides*. Initially, we successfully induced a cirrhosis model using CCL_4_ and confirmed PVT formation using ultrasound. Supplementation with *Bacteroides* significantly reduced PVT volume as well as increased blood flow (Figures 6A,B). We examined fibrinogen, D-Dimer, and P-Selectin in plasma by ELISA and showed that they were significantly elevated in the CCL_4_ group and decreased after *Bacteroides* treatment (Figures 6C-E). These data indicate that *Bacteroides* supplementation ameliorates CCl_4_-induced PVT in Wistar rats.

## Discussion

One of the characteristics of cirrhosis is gut dysbiosis, which is a reduction in gut microbiota diversity with a relative or absolute increase in opportunistic species (Trebicka et al., 2021). PVT is a serious complication of late-stage cirrhosis, and our data suggest that a correlation between the gut microbiota and PVT in LC, gut dysbiosis may be involved in its occurrence, which can affect PVT progression through the following mechanisms: First, translocation of bacteria or bacterial products (such as LPS) into the portal vein accounts for endotoxemia (Fukui, 2015). Hyperendotoxemia aggravates the inflammatory response in the liver (Baffy, 2019) and also leads to abnormal coagulation and PVT formation (Violi et al., 1995). The vasoactive substances released by some gut bacteria may promote visceral vasodilation, resulting in arteriolar vasodilation, reduced systemic vascular resistance, and increased portal inflow, further contributing to portal hypertension, which is a key factor in PVT formation (Wiest and Groszmann, 2002).

Our study focused on changes at the genus level in the gut microbiota and identified distinctive differences in the proportions of several bacterial taxa in fecal samples collected from patients with LC and PVT. Patients with LC typically exhibit gut dysbiosis, which is characterized by an overgrowth of gram-negative bacterial taxa (Chen et al., 2011; Wang et al., 2021). Similarly, in this study, we found that the gut microbiota of patients with cirrhosis mainly comprised gram-negative bacteria. However, in cirrhosis with PVT, the diversity of the gut microbiota increased significantly, and the abundance of pathogenic bacteria such as *Anaerostipes* and *Pseudomonas* increased. The abundance of probiotic microorganisms such as *Bacteroides* and *Weissella* decreased significantly in cirrhosis patients with PVT. This difference in microbiota also led to significant differences in metabolism, which could lead to large differences in metabolites; however, relevant tests were not performed in this study.

The abundance of *Anaerostipes* is increased in a variety of diseases, such as endometrial cancer (Walther-António et al., 2016), encephalitis (Gong et al., 2021), and depression (Barandouzi et al., 2020), and it is involved in disease progression, mainly through immune regulation. *Pseudomonas* is a typical pathogenic bacterium, especially *Pseudomonas aeruginosa*, which causes many infectious diseases. Similarly, our data also confirms that their abundance increases in the PVT group, these pathogenic bacteria induce an inflammatory response that aggravates liver damage by releasing inflammatory cytokines and LPS.

The *Bacteroides*, a type of intestinal probiotic, our research confirms that was significantly less abundant in the LC with PVT. We effectively reversed PVT formation by supplementing Carbon tetrachloride-constructed rat models of cirrhosis with *Bacteroides*, which surfaced that it may be a potential treatment for PVT. Although the specific mechanisms we have not explored in depth, some studies have reported that *Bacteroides* involved in several functions within the human gut bacteriome, such as the metabolism of polysaccharides and oligosaccharides. Additionally, they aid in the provision of nutrition and vitamins to both the host and other gut microbial residents (Zafar and Saier, 2021). More importantly, it can play an important role in tumors and inflammatory diseases by regulating inflammatory responses (Tan et al., 2019). The other important probiotics microorganism, such as *Weissella*, plays a beneficial role by anti-inflammatory and immunomodulatory properties (Ahmed et al., 2022). The *Weissella* supplementation has been shown to have a reversal effect on hepatic fibrosis (Jantararussamee et al., 2021) and fatty liver (Choi et al., 2021). Our results suggest that these probiotics are significantly decreased in LC with PVT, which we speculate plays an important role in the occurrence of PVT.

Coagulation disorders are integral components of liver cirrhosis and are more pronounced in PVT. Consistent with previous reports, D-Dimer levels were significantly elevated in PVT in our study, which promoted thrombus formation (O'Leary et al., 2019; Pan et al., 2022). Interestingly, our results demonstrated a clear correlation between the presence of various microbiome genera and coagulation disorders. The imbalance of inflammatory states and metabolites caused by a large number of gram-negative bacteria and anaerobic bacteria leads to activation of the coagulation cascade (Hasan et al., 2020), which may mediate PVT pathogenesis.

## Conclusion

The composition of gut microbiome shows significant variation between LC patients with and without PVT, and certain genera exhibit differing levels of abundance that are correlated with the coagulation function’s clinical characteristics. We emphasizing modulating the gut microbiota, especially *Bacteroides*, may be a promising therapeutic approach to reduce the progression of PVT in LC.

## Data availability statement

The data presented in the study are deposited in the NCBI repository, accession number PRJNA967488.

## Ethics statement

The studies involving humans and animal study were approved by The Ethics Committee of Sichuan Provincial People’s Hospital. The studies were conducted in accordance with the local legislation and institutional requirements. The participants provided their written informed consent to participate in this study.

## Author contributions

X-yH, Y-hZ, and DZ contributed to conception and design of the study. S-yY, LL, and TM organized the database. S-yY and X-yH performed the statistical analysis. X-yH wrote the first draft of the manuscript. RH, LY, Z-mL, and DZ wrote sections of the manuscript. All authors contributed to the article and approved the submitted version.

## Abbreviations

PVT: Portal vein thrombosis
LC: Liver cirrhosis
